# VIGLA-M: visual gene expression data analytics

**DOI:** 10.1186/s12859-019-2695-7

**Published:** 2019-04-18

**Authors:** Ismael Navas-Delgado, José García-Nieto, Esteban López-Camacho, Maciej Rybinski, Rocio Lavado, Miguel Ángel Berciano Guerrero, José F. Aldana-Montes

**Affiliations:** 1 Khaos Research, Universidad de Málaga, Málaga, Spain, Arquitecto Francisco Peñalosa 18, Málaga, 29071 Spain; 2 Unidad de Oncología Intercentros, Hospitales Univesitarios Regional y Virgen de la Victoria de Málaga, Instituto de Investigaciones Biomédicas (IBIMA), Málaga, Spain, Málaga, Spain

**Keywords:** Gene Expression Level Analysis, Nanostring Immune Profiling Panel, Metastatic Melanoma

## Abstract

**Background:**

The analysis of gene expression levels is used in many clinical studies to know how patients evolve or to find new genetic biomarkers that could help in clinical decision making. However, the techniques and software available for these analyses are not intended for physicians, but for geneticists. However, enabling physicians to make initial discoveries on these data would benefit in the clinical assay development.

**Results:**

Melanoma is a highly immunogenic tumor. Therefore, in recent years physicians have incorporated immune system altering drugs into their therapeutic arsenal against this disease, revolutionizing the treatment of patients with an advanced stage of the cancer. This has led us to explore and deepen our knowledge of the immunology surrounding melanoma, in order to optimize the approach. Within this project we have developed a database for collecting relevant clinical information for melanoma patients, including the storage of patient gene expression levels obtained from the NanoString platform (several samples are taken from each patient). The Immune Profiling Panel is used in this case. This database is being exploited through the analysis of the different expression profiles of the patients. This analysis is being done with Python, and a parallel version of the algorithms is available with Apache Spark to provide scalability as needed.

**Conclusions:**

VIGLA-M, the visual analysis tool for gene expression levels in melanoma patients is available at http://khaos.uma.es/melanoma/. The platform with real clinical data can be accessed with a demo user account, *physician*, using password *physician_test_7634* (if you encounter any problems, contact us at this email address: mailto: khaos@lcc.uma.es). The initial results of the analysis of gene expression levels using these tools are providing first insights into the patients’ evolution. These results are promising, but larger scale tests must be developed once new patients have been sequenced, to discover new genetic biomarkers.

## Background

The analysis of the gene expression changes and their correlation with clinical changes can be developed using the tools provided by those companies that currently provide gene expression platforms, such as NanoString. The analysis of gene expression levels is a very important problem when studying how patients evolve, and looking for a way to find new genetic biomarkers helping in clinical decision making. Current solutions are based on desktop tools designed for use by geneticists. However, it is the clinicians who are in direct contact with the patients, so their ability to access such data in prior analyses could prove highly beneficial.

NanoStringDiff [1] is an R package for differential expression analysis of NanoString nCounter data, whose main function for differential analysis is glm.LRT. The use of this tool requires knowledge of R programming language.

NanoStringNorm [2] provides R functions for preprocessing methods proposed by NanoString in addition to an extensible plug and play framework to incorporate other methods. This tool also requires R knowledge from its users.

nCounter [3] is the advanced analytical tool provided by NanoString Technologies, Inc. This tool provides different analytical tools for gene expression data. It gives high quality results, but it has been designed for use by geneticists and so, it has a steep learning curve for physicians and clinical researchers. These users hope to make initial discoveries based on the experimental results, which normally requires help of an experienced nCounter user.

nSolver [4] is the simplest analytical tool provided by NanoString Technologies, Inc. This tool provides a desktop user interface where users can import reporter code count (RCC) files for different analyses. Each RCC file contains results of a single flow cell of the cartridge. Each RCC file contains the number of counts for each target mRNA molecule (measured within one sample). A separate RCC file is created for each flow cell (or sample) in the cartridge. Although easier to use than nCounter, the tool still requires some learning effort, even for simple data analyses. In addition, the lack of some parametrization options makes it difficult to extract relevant information. As an example, a simple heat map analysis does not provide a way of filtering out those genes with lower variability.

NanoStriDE [5] allowed biologists to take raw data produced by a NanoString nCounter analysis system and easily interpret differential expression analysis of this data represented through a heatmap. However, the system was not easy to use, and in some cases clinicians required biostatisticians to help them with these tasks.

The analysis of gene expression levels is a complex problem that is being studied from different perspectives. Thus, [6] presents an approach to parallelize the TriCluster algorithm to be used in this context. In [7], another parallelization approach is proposed for efficient large-scale transcriptome data analysis.

The gene expression platform used in this work is NanoString, which provides nCounter. However, nCounter has some limitations as the type of analysis is restricted to a predefined set, and the introduction of clinical features is a complex task. This project presents an approach to collect the clinical information using a structured database and a web user interface to introduce this information, including the results of the gene expression measurements, as a move a step closer towards the clinicians, in comparison to the nCounter tool.

This paper presents VIGLA-M, an analytics tool to provide clinical researchers with early insights into the gene expression samples of a patient (discovering changes in their gene expression levels) or sets of patients (discovering patient clusters with a possible clinical correlation). Once the physicians or clinical researchers find relevant information in these data, geneticists can build on the preliminary analysis using, for instance, nCounter.

The tool proposed in this paper has been tested on a small set of patients of metastatic melanoma patients, but it is intended to support similar cases in other diseases where gene expression variation analysis would help in discovering new biomarkers.

At present, immunotherapy for metastatic melanoma is based on stimulating an individual’s own immune system, in most cases through the use of specific monoclonal antibodies. The use of immunotherapy has meant that many patients with melanoma have survived and, therefore, it constitutes a promising present and future treatment in this field [8–12].

At the same time, drugs have been developed targeting specific mutations, i.e. BRAF (BRAF is a human gene that encodes a protein called B-Raf), resulting in significant responses in tumor regression (set up in this clinical study for 18 months), as well as a higher percentage of long-term survivors [13–16]. These significant responses are triggered by a massive process of antigens creation, that is needed to reignite a state of immunocompetence in the patient. It also leads to the activation of biomarkers that could guide response prediction.

Unfortunately, the recent interest in developing drugs against melanoma has not been accompanied by the identification of biomarkers that are predictive of response or toxicity to them. Although preliminary data determined immunohistochemistry was useful in the tumor expression of the PDL-1 protein, subsequent work has failed to validate its predictive usefulness. In fact, one of the main problems in the study of the immune system is its plasticity and adaptive nature. Tumor infiltrating lymphocytes (TIL), another possible postulated and non-predictive marker, validated that PDL-1 expression can fluctuate within short time frames and its expression changes when biopsies are performed serially over time on the same tumor [17]. Therefore, more biomarkers are needed to optimize the sequences of treatments against melanoma. For these reasons, our work aims to combine both concepts: the use of therapy directed against the BRAF mutation and the reactivation of acquired immunity against melanoma.

Therefore, it would be useful to look for these changes in blood biomarkers in relation to immunological mechanisms to identify predictive markers of response to new treatments, in order to identify long-term survivors with targeted therapy. Unfortunately, many patients will not benefit from these treatments, as they are difficult to fund in terms of human and economic expenditure. However, this project seems to be an essential first step and the finding of these biomarkers is yet to be explored.

Additionally, we have pursued a first approach to parallelize the algorithms using Big Data technologies (Apache Spark), to support large scale clinical assays. At this stage, VIGLA-M has been tested for a clinical assay with metastatic melanoma patients.

## Implementation

In this section we present the three main functionalities that have been developed in the VIGLA-M project: clinical data collection, gene expression data collection and gene expression level data analysis and visualization.

**Fig. 1:**
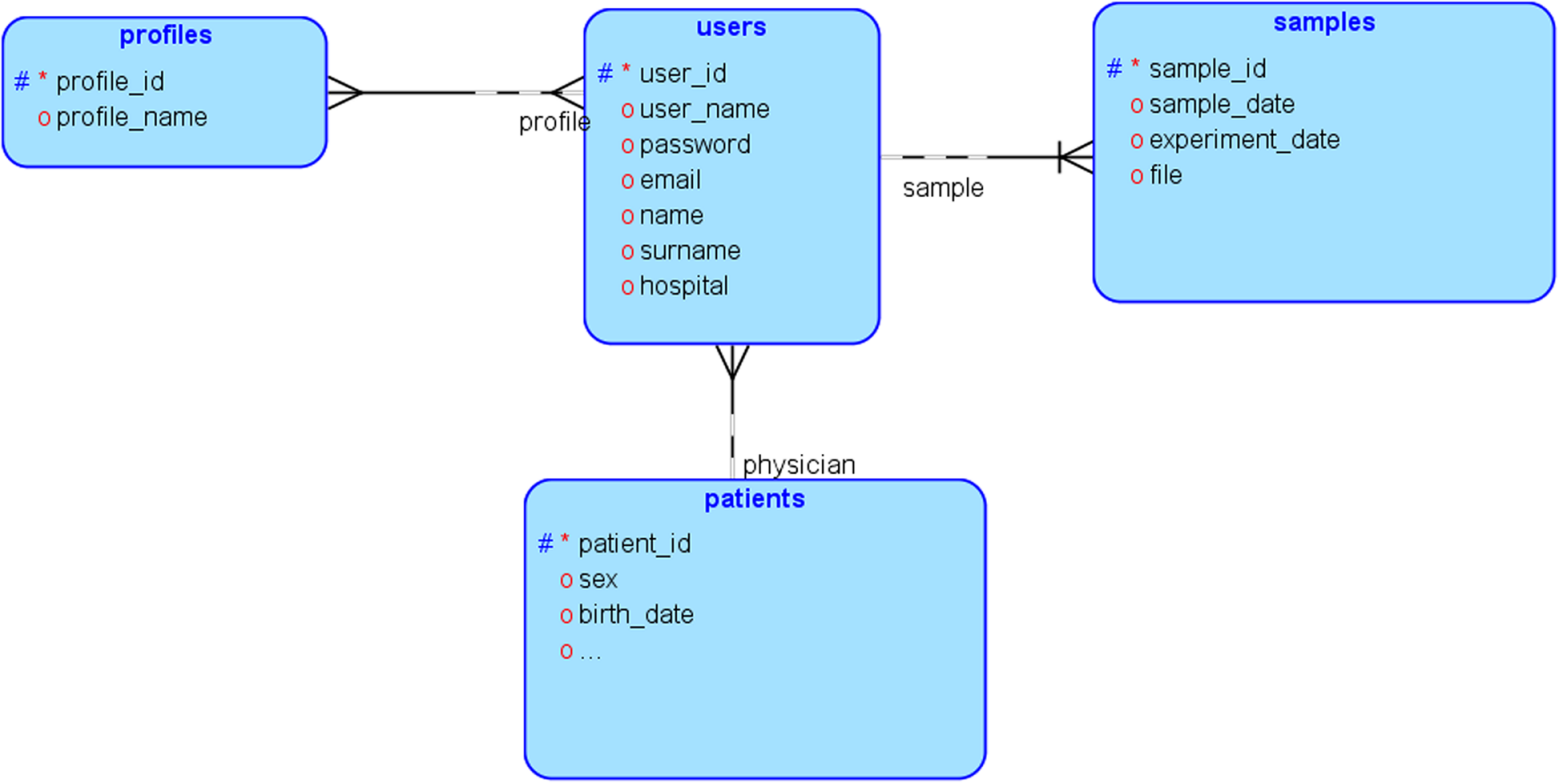
Extract of the database. This figure has been produced using SQL Developer Data Modeler (Oracle Database Design Tool). Users are connected with one or more profiles (user types), meanwhile profiles can also be connected with several users. Samples cannot exist without a user, and a user can have several samples. Patients are linked to a user with a profile of type “physician”

To collect the required data, we have developed a structured database (using Oracle 11g Express Edition) to store clinical and gene expression data. Figure 1 shows part of the database schema, which is designed to store: a minimal set of personal patient data (gender, date of birth, medical records, medication, diagnosis date, metastasis diagnosis date, etc.); the response to the treatment; the cancer’s progress, including the different visits to the hospital and the blood test values at each visit; the BRAF mutation; primary location of the cancer, and the files associated with the gene expression analysis.

The clinical information is collected by the patient’s physician (previously registered and accepted in the clinical assay by the clinical project coordinator). Thus, the effort of collecting the data is shared by all the physicians participating in the project (clinical assay).

The files obtained from the gene expression assays are loaded by the biologists (previously registered in the system). These files are obtained from the NanoString platform. The NanoString platform can analyze 12 samples in each cartridge. As a result, we obtain 12 RCC files with the gene counts for each of the gene panels. Each file is associated with the gene expression of a patient at a specific stage of treatment. These files are locally stored with a patient code, in addition to being annotated with the patient code from the project database, the sample collection date and the Nanostring experiment date. Then, the biologist uploads the files attached to the patient profile, along with other metadata.

We have used the Immune Profiling Panel Nanostring ^*TM*^ (770 genes), as it has been specifically designed for cancer projects where immune aspects are studied. This panel includes 24 different immune cell types, common checkpoint inhibitors, CT antigens, and genes covering both, adaptive and innate, immune responses.

### Data normalization

Using the gene expression files, a set of analytic functionalities has been developed to discover patterns in the change of the gene expression levels. However, these files need to be preprocessed, as NanoString returns the flat counts of the gene expression levels. The pre-processing is done to normalize the counts according to a set of control measures. The preprocessing is described in NanoString guidelines [18], and it can be summarized with the following steps:

- Step 1. Generation of quality control flags (binding density, positive control linearity, limit of detection).
- Step 2. Background correction using the negative control samples.
- Step 3. Calculation of lane-specific scaling factors, based on the *housekeeping* gene set.
- Step 4. Adjusting the flat counts with the lane-specific scaling factor.

Step 1 is to ensure that the end user is informed about the quality of the samples. The binding density is included in the NanoString output, therefore it is simply read from the sample file. Positive control linearity is the process of evaluating whether the flat counts obtained for synthetic-positive-control samples maintain the expected linear relationship resulting from their known amounts. Therefore, linearity is calculated as the *r*^2^ value between the NanoString values and known values. The limit of detection is evaluated by comparing one of the positive controls and its distance from the background. The flags are directly passed on to the visualization module, so that the end user gets notified of any possible quality problems.

Step 2 is done through background thresholding – setting the values of flat counts that fall below a certain threshold value to this threshold value. The threshold value is calculated as a two standard deviations of the negative control sample values over the geometric mean of negative control sample values.

Step 3 is carried out along the same lines as those presented in [19]. A subset of at least 3 most stably-expressed housekeepers is found and then used to calculate the scaling factors.

Step 4 is done simply by multiplying the flat counts with the scaling factors calculated in Step 3. The method for finding the most stable genes is illustrated with the following pseudocode snippet:


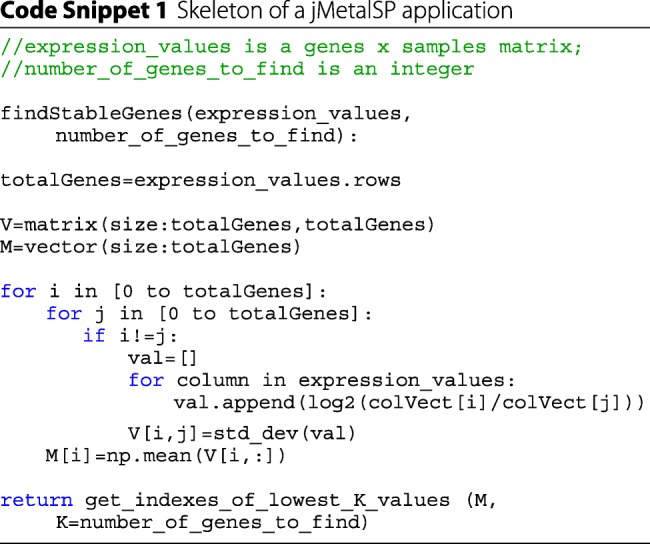


Subsequently, the lane-specific normalization factors are calculated as the inverse of the geometric mean of the in-lane housekeeper counts multiplied by the average value of these geometric means (across all lanes).

### Gene filtering

The Immune Profiling Panel Nanostring ^*TM*^ contains 770 genes, so any visualization including all these genes would be difficult to explore for the end-users. For this reason, the developed tool includes a filtering module able to filter out those genes that do not change (or change less than the rest) over the analyzed samples.

The filter is also based on the geNorm method. Specifically, geNorm postulates finding *n* (>3) most stable housekeeping genes. For filtering, we use the same model to find *k* (parameter specified by the user, e.g. 100) least stable genes, i.e., the ones that display most variation (see the code snippet).

## Results

**Fig. 2:**
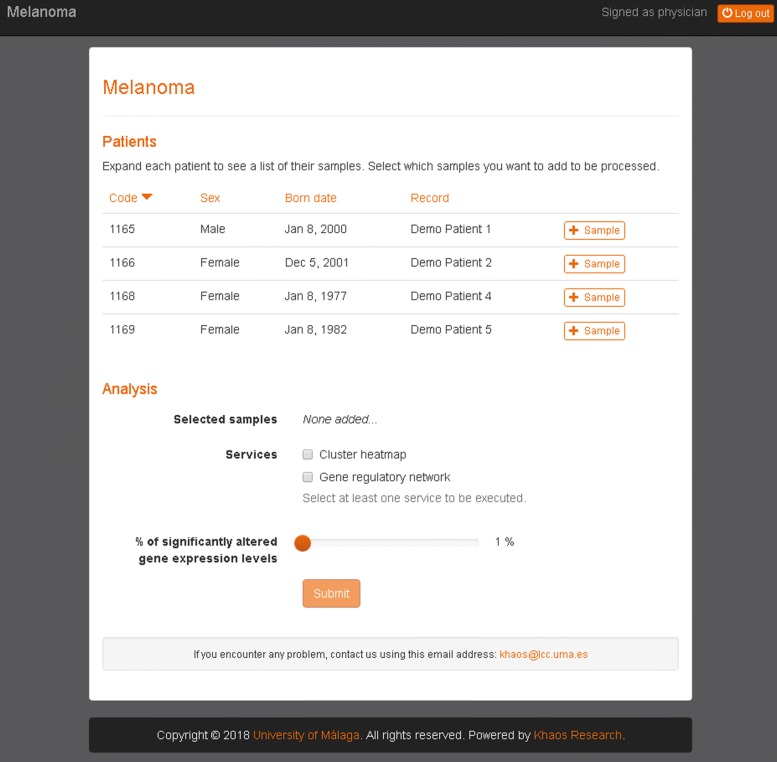
List of patients for a demo user. For each patient the user can click on Sample to add one or more samples for them

To enable users to exploit the processed data we have developed VIGLA-M, a web service for accessing and analyzing these data. Registered users can log on to the tool and browse through the data of their patients. The tool available at http://khaos.uma.es/melanoma has a demo user with demo patients with real gene-expression data. User and password for demo access are: physician and physician_test_7634 respectively. Physicians can only access their patients’ data, biologists can only access gene expression data that they have uploaded, and clinical assay administrators can access all the data (see Fig. 2 for the demo user).

**Fig. 3:**
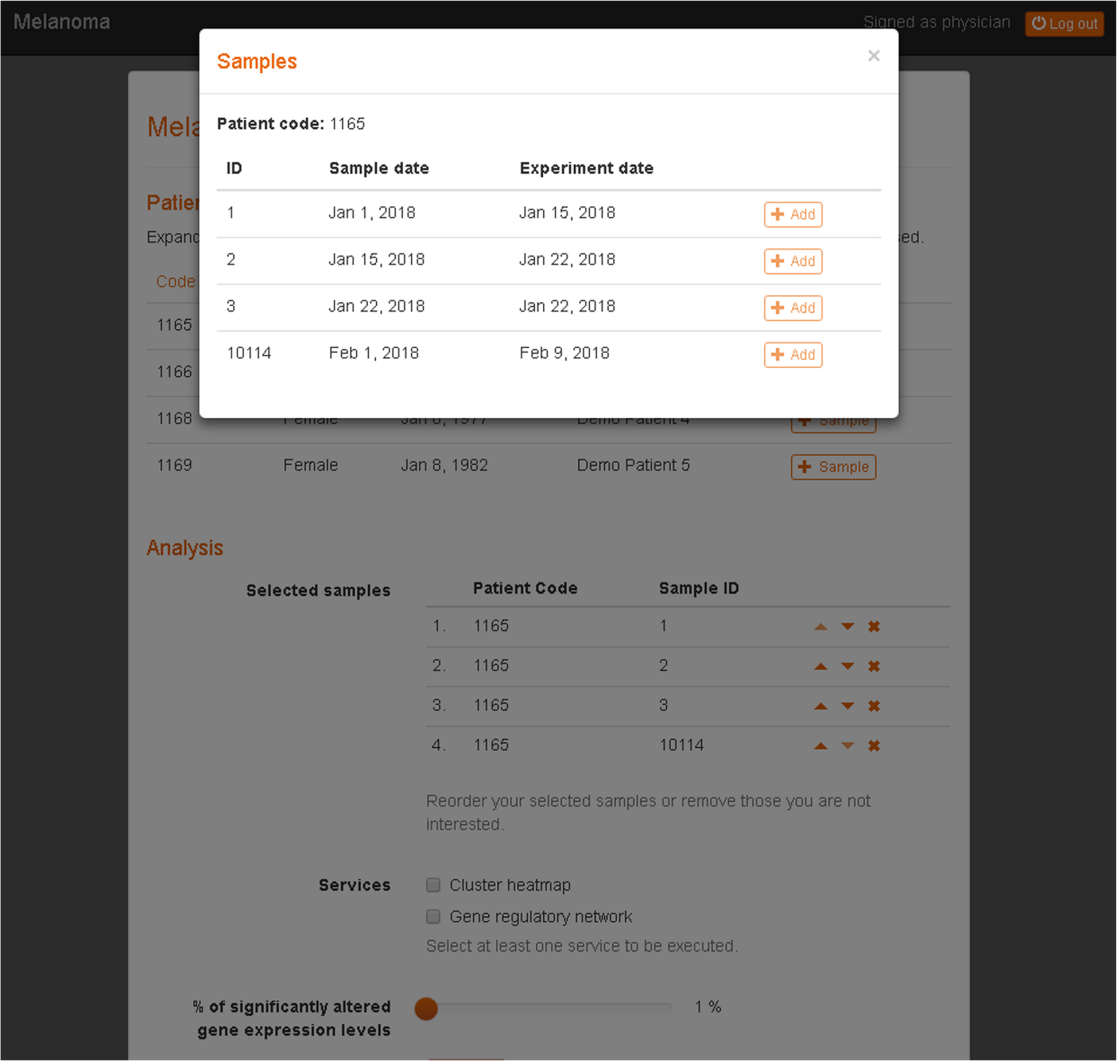
Sample selection from the available patient data. The user can select one or more samples from the same patient

Users can select different gene expression samples from one or more patients to start the gene expression analysis (see Fig. 3 for the demo user).

**Fig. 4:**
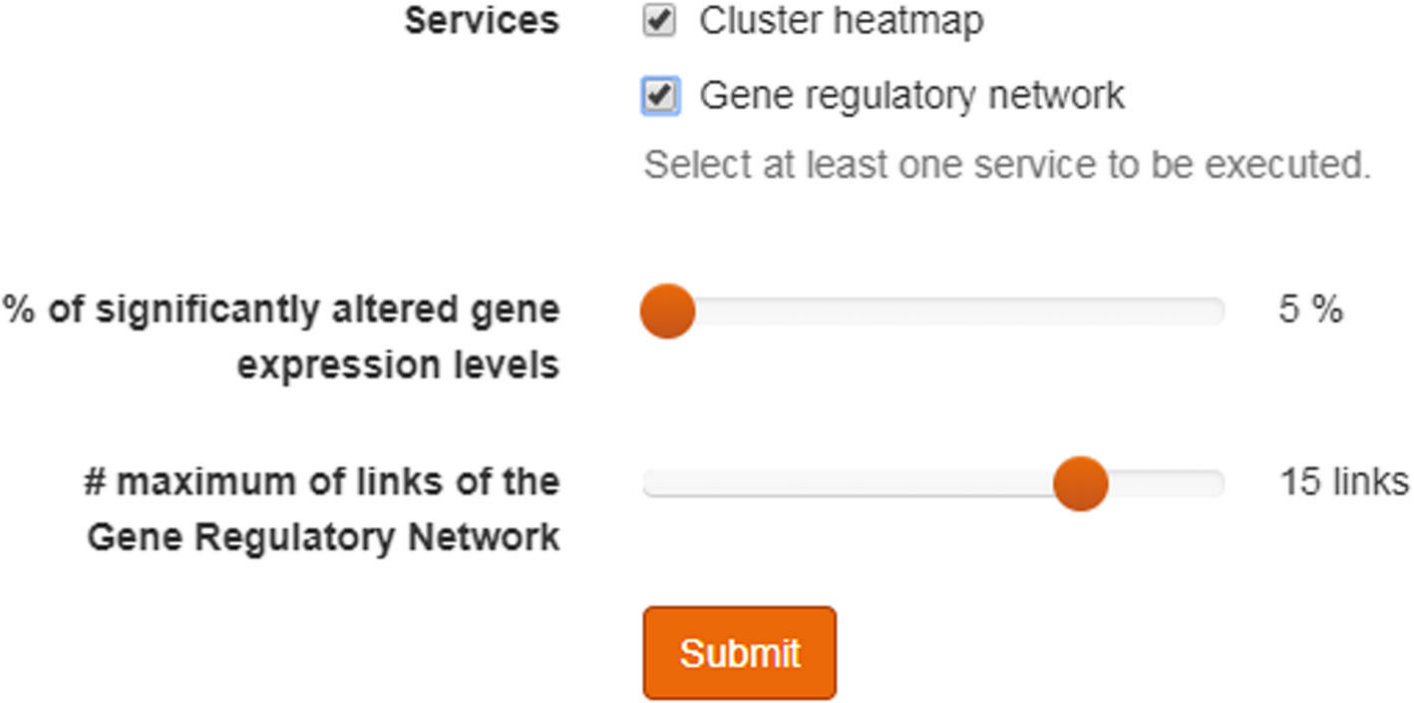
Selection slide for controlling the size of the gene regulatory network. Once the user has select one or more samples from one or more patients, the user can select between the different analytical tools. The figure shows the two current options: Heat Maps and Gene Regulatory Networks. The slides enable users to determine which section of the data will be included in the graphical representation of the results
  To generate the network, two well-known algorithms [20] can be used. Both implementations are provided in the *arboretum* Python package (https://arboreto.readthedocs.io/en/latest/, namely: **GRNBoost2**, a fast algorithm based on stochastic Gradient Boosting Machine regression with early-stopping regularization; and **GENIE3**, the popular classic GRN inference algorithm based on Random Forest (RF) or ExtraTrees (ET) regression. This choice option will enable physicians to inspect different learning models for inferring the underlying GRNs.

Therefore, using the normalized gene expression values of the most variable genes, the proposed tool is designed to perform two main functionalities for data analysis and visualization:

- *Heat Maps*. A heat map provides a graphical representation of data stored as a matrix. The values included in the matrix are represented as colors. The tool provides a sliding element to set a parameter for extracting only the most variable gene expression levels (as a percentage of the total number of genes). It is worth mentioning that gene expression levels are standardized by calculating z-scores, hence providing balanced values before the visualization step. In addition to this, hierarchical clustering is computed to generate groups of both, genes and experiments. This will provide additional information to physicians about how samples can be organized.
- *Inference of Gene Regulatory Network*. Gene Regulatory Networks (GRNs) can be reconstructed from a time series of gene expression data. This information is of particular clinical interest because it can serve to detect genes that are interacting and so, detect new pharmacological targets. The tool provides a sliding element to set the parameter for representing only the most relevant (*n*) links of the network (as large networks will be difficult to interpret). This filter keeps the nodes involved in the *n* relationships with highest weights (see Fig. 4).

### Data analysis through heat map visualization

The use of heat maps in VIGLA-M provides a flexible way of producing different types of analysis with respect to the selected gene expression data and their order. Thus, depending on these features, it is possible to produce four different representations to help clinicians analyze the expression results:

- Type I. Comparison of several samples for a single patient (patient evolution). The visualization of the expression change in a patient enables the individual analysis of the patient data with a high degree of detail.
- Type II. Comparison of the basal status of the patients (classification of the patients depending on the initial expression profile). This visualization enables the analysis of the gene expression profiles to allow users to analyze those patients with a specific pattern. For example, a user could select those patients with certain genes with a lower expression than the rest of the patients, to later explore the possible expression growth.
- Type III. Comparison of the second sample of the patients (classification of the patients according to their expression after the evolution). This visualization also allows comparisons of patients after treatment.
- Type IV. Comparison of the patients including both samples (profiling on the patient evolution in the gene expression). This visualization type is the most complete one provided by this tool. It enables the classification and visualization of differences between patients in the evolution of the gene expression. In this case, the patient samples are ordered showing first the initial sample of all the patients, and then, the results for the second sample.

**Fig. 5:**
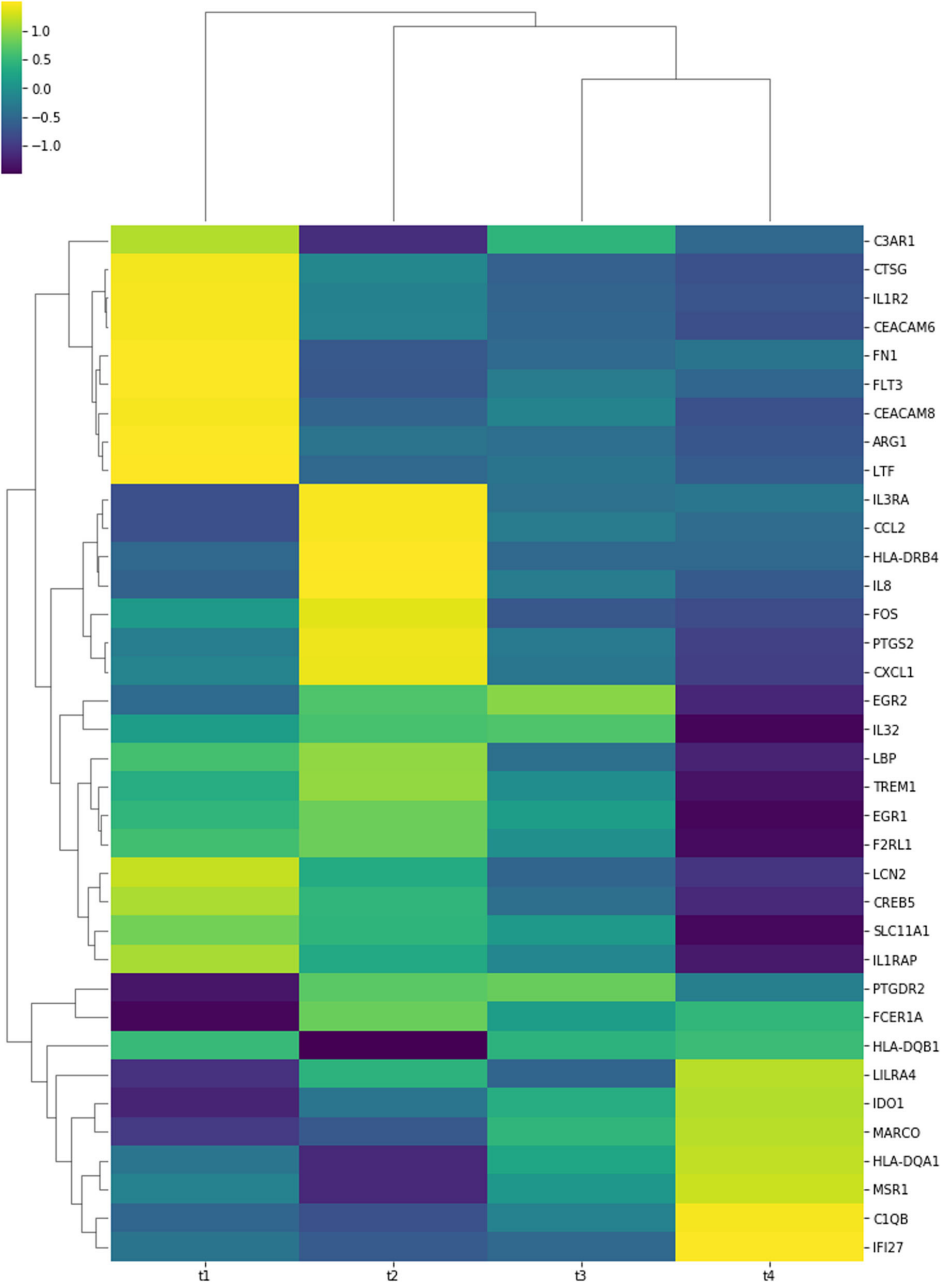
Heatmap with hierarchical clustering generated for patient with allergic reaction. The X axis shows the four different samples for this patient over a stretch of time (t1: sample before the treatment; t2: sample after the treatment with BRAF inhibitors; t3: sample just after the allergic reaction; t4: sample after the allergic reaction disappears). The Y axis show the genes that had more changes in this time series

Figure 5 shows a clinical case for Type I. This case can be replicated using the samples from Demo Patient 1. This patient suffered an allergic reaction to the treatment for the melanoma cancer. The first graphical representation is a heat map with hierarchical clustering according to both, genes and assays. As input this heat map only needs a set of ordered gene expression files.

The heat map shows the most significant changes that have occurred in the expression of some genes of immune function, at different times of the treatment for metastatic melanoma. Prior to treatment, there was an activation of several genes, whose activity may be associated with increased tumor replication. Thus, there are complement (C3AR1), tyrosine kinase (FLT3 or CD135, therapeutic target of drugs such as sorafenib or sunitinib), interleukins (IL1R2) and adhesion molecules (CEACAM6) receptors or fibronectin (FN1), which are usually activated in many tumors. With BRAF inhibitors, these genes reduce their expression, showing the efficacy of this treatment in the subject under study.

With the allergic reaction to the BRAF inhibitor other genes were activated, which were initially not active, such as IL3RA, CCL2, HLA-DRB4, IL8, FOS, PTGS2, CXCL1, and which were subsequently corrected with the use of drug sensitization with corticosteroids and gradual exposure to the drugs. The activation of these genes can improve the immune response, through the interleukins (IL3 and IL8) or the better presentation of antigens (HLADRB4), and so is able to produce a good response to the drugs.

**Fig. 6:**
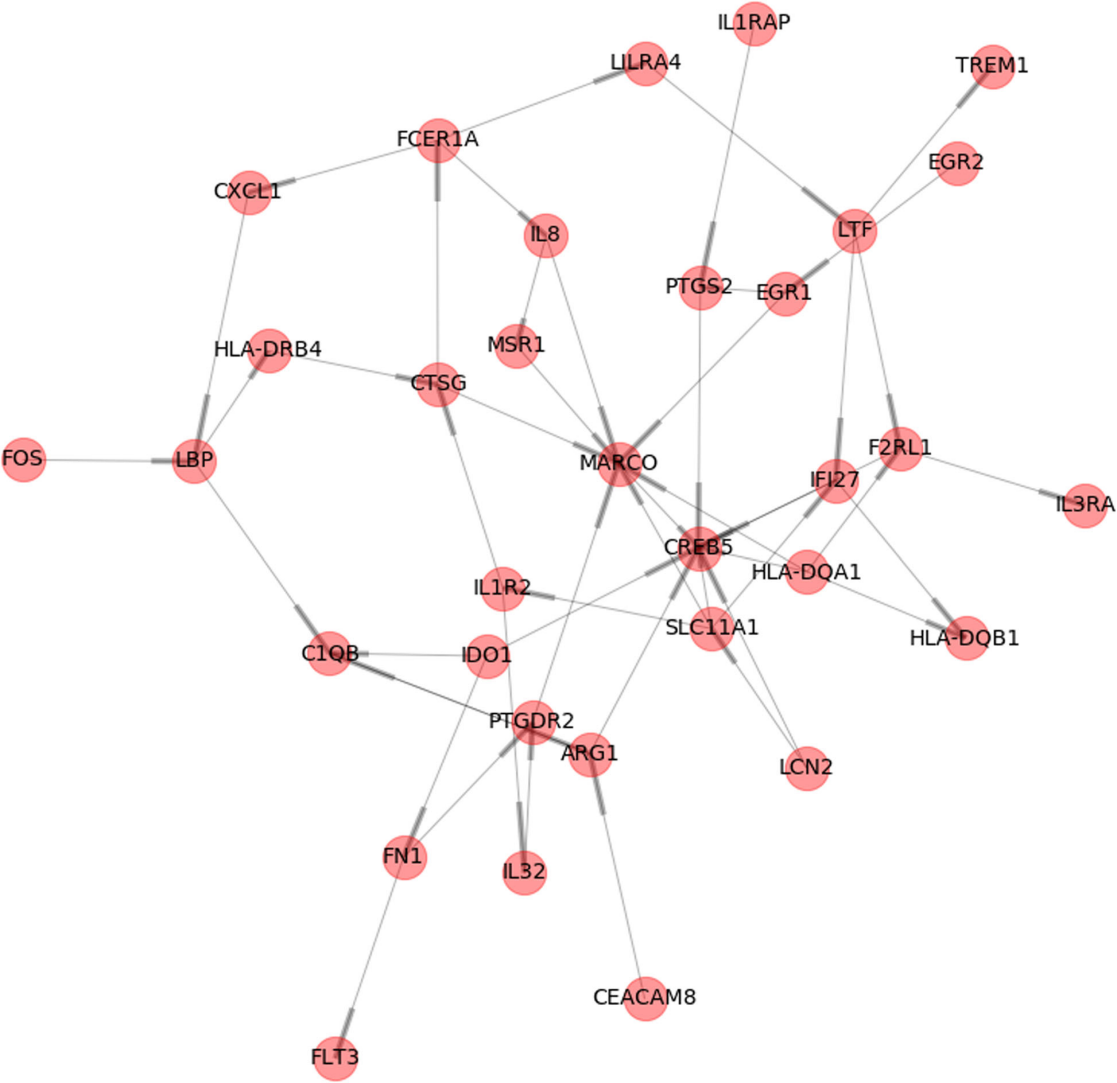
Gene regulatory network obtained from GRNBoost2 algorithm for patient allergic reaction. Thicker part of the lines shows the direction of the arc (that is, the regulatory effect). For example, on the left we can see that FOS regulates LBP. The longer the line, the longer the regulatory strength

Finally, the heat map also shows how, over time, there are several genes that are activated (LILRA4, IDO1, MARCO, HLA-DQA1, MSR1, C1OB, IF27) and others that are deactivated (EGR2 and 1, IL32, EBP, TREM1, F2RL1, LCN2, CREB5, SLC11A1, IL1TRAP). Some of these genes are the key to immunotherapy resistance based on inhibitors of immunocheckpoints, so they can also be key in the immune response induced by targeted therapy against BRAF. Thus, IDO not only appears to be a key factor in the gene interactions tree (see Fig. 6), but it is a novel target. The inhibition of this enzyme is promising given the results of early phase drug development [21].

### Data analysis through gene regulatory network reconstruction

Inferring the interaction network of genes is a fundamental step towards understanding how a cell or an organism will respond to its environment. Its use in clinical assays would be of interest as it provides the insight on how different genes affect the expression of other related (or not) genes. Following the example of the patient with an allergic reaction, we have used the tool to reconstruct the Gene Regulatory Network.

The resulting network is shown in Fig. 6, which visualizes how activated and deactivated genes (according to the previous analysis) take part in the regulatory graph with strong interactions between them. In this figure, the thicker part of the lines in edges show the direction of the arcs (in form of arrows), thus indicating the regulatory effect. This differentiates GRN from gene co-expression networks, in which the direction of edges is overlooked. In Fig. 6, it can be clearly seen that genes like MARCO, C1OB, IF27, and CREB5 act as transcription factors that play important role in the regulation phase of the network. Strong interactions are registered for the following interactions: IL8 →MSR1, MSR1 →MARCO, MARCO →CREB5, and IF27 →CREB5. This could shed light on the inmunotherapy response of the organism, so the clinician is now supported with additional arguments in adjusting the treatment process.

## Discussion

The complexity of nSolver and nCounter means that physicians are reluctant to explore the gene expression data, and they will analyze only high quality results obtained by geneticists using these tools. These tools are quite useful for reaching the main goals of these clinical research projects, but at the end of the clinical assay. In this paper, we present a tool that gives the physicians the possibility to make initial explorations of these data with ease, gaining first insights that can later be corroborated by the geneticists.

The initial evaluation shows that for small datasets this tool is powerful enough, although the initial experiments indicate that the growth in the number of samples could make it unfeasible. For this reason, a first prototype using Apache Spark has been developed to prepare this tool to scale to larger datasets. Thus, the internal calculations can be executed in a Spark cluster with the required size (depending on the dataset size), scaling horizontally.

At the same time, the connection (by design) with clinical variables, will enable the development of a new set of tools for searching correlations between the gene expression analysis and the patient evolution.

## Conclusions

This paper has presented VIGLA-M, a useful tool in form of web service for use in clinical assays, to enable physicians the exploration of gene expression data. This tool is being tested in the context of a clinical assay with metastatic melanoma patients, which are currently treated with immunotherapy. First results obtained with the assist of VIGLA-M have been reported in several clinical forums [22, 23].

The use in a real scenario, where first clinical insights are being obtained, has served to validate this service. Even though this is not a scenario with real Big Data features, a first prototype using Apache Spark has been developed.

The proposed service is conceived to serve as a more directly usable complement to nSolver and nCounter, although it is also designed to include other clinical variables that will be exploited in future work.

## Availability and requirements

**Project name:** VIGLA-M: visual gene expression data analytics

**Project home page:** http://khaos.uma.es/melanoma/

**Operating system(s):** Platform independent

**Programming language:** Java

**License:** GNU GPL

**Any restrictions to use by non-academics:** licence not needed

## Abbreviations

ET: ExtraTrees algorithm
RCC: Format used by NanoString Technologies, Inc. for the files with expression data
BRAF: It is a human gene that encodes a protein called B-Raf
GRN: Gene regulatory network
RF: Random forest algorithm
